# Features of gut microbiota and short-chain fatty acids in patients with first-episode depression and their relationship with the clinical symptoms

**DOI:** 10.3389/fpsyg.2023.1088268

**Published:** 2023-04-24

**Authors:** Shuhan Yu, Lan Wang, Xinyang Jing, Yujing Wang, Cuixia An

**Affiliations:** Mental Health Center, The First Hospital of Hebei Medical University, Hebei Clinical Research Center for Mental Disorders and Institute of Mental Health, Hebei Technical Innovation Center for Mental Health Assessment and Intervention, Shijiazhuang, China

**Keywords:** major depressive disorder (MDD), first episode, intestinal flora, short-chain fatty acids, correlation analysis

## Abstract

**Objective:**

To compare the differences in gut microbiota and short-chain fatty acids (SCFAs; metabolites of gut microbiota) in the serum of patients with first-episode depression and the healthy population and to analyze the relationship between gut microbiota and metabolite SCFAs and the clinical symptoms of major depressive disorder (MDD).

**Methods:**

A total of 45 patients with first-episode depression and 22 healthy volunteers were chosen to complete relevant scale evaluations, and feces samples and venous blood samples were collected. The 16S RNA method was used to analyze the intestinal microflora and the characteristics of serum SCFAs detection by ELISA kit, as well as the intestinal flora, SCFAs content and their correlation with MDD clinical indicators.

**Results:**

The abundance of *Akkermansia*, *Megamonas*, *Prevotellaceae NK3B31 group*, and butyrate-producing bacteria, *Lachnospira*, *Subdoligranulum*, *Blautia*, and *Dialister*, and acetate-producing bacteria, *Streptococcus*, in the gut microbiota of the MDD group was lower than that in the control (C) group. The abundance of *Parasutterella* in the MDD group was higher than that in the C group. *Dialister* negatively correlated with all measured clinical symptoms (*r* < 0, *P* < 0.05). The serum SCFA content in the MDD group was higher than that in the C group, and the content positively correlated with the Hamilton anxiety scale scores (*r* = 0.584, *P* < 0.05).

**Conclusion:**

The results demonstrated that the MDD group differed from the C group in terms of gut microbiota and SCFAs in the serum and that the change in certain intestinal bacteria might participate in the pathogenic mechanism of MDD.

## 1. Introduction

Major depressive disorder (MDD) is a common mental disorder with various causes that includes significant and chronic depressive syndromes. The main symptoms are depression, loss of interest and lack of energy, accompanied by other cognitive, physiological and behavioral symptoms. The lifetime prevalence of MDD is up to 10.8–20.6% (World Health Organization [WHO], 2017), and according to an analysis in the Global Burden of Disease Study 2017, MDD is the third most common cause of the increase in years lived with disability. The etiology of MDD is not fully clear, and many studies suggest it may be associated with genetic factors, changes in neurotransmitter levels, disorders of the neurologic function channel, neuroendocrine disorders, and psychological and social factors (Brown et al., 2015). In recent years, an increasing number of studies have demonstrated that the pathogenic mechanism of MDD might be closely related to a disorder of the gut microbiota (Halverson and Alagiakrishnan, 2020). Short-chain fatty acids (SCFAs), which include butyrate, propionate, and acetate, are essential metabolites for the activity of intestinal microorganisms (Dinan and Cryan, 2017), and they can play a central role through G-protein-coupled receptors in affecting emotional states and recognition, indicating a correlation between SCFAs and the pathogenic mechanism of MDD. This viewpoint was confirmed in an animal experiment (Li J. et al., 2018). Sterile mice showed depression after transplantation of fecal microorganisms from patients with depression (Li B. et al., 2018). A meta-analysis (Sanada et al., 2020) of the human study included eight studies on the differences of intestinal microflora in depression, and the results showed that the results of various studies were not consistent at the phylum level. At the family level, at least two studies showed that the number of patients with depression was decreased, while the number of actinomycetes was increased. At the genus level, at least two studies found that the number of patients with depression was faecalis, ruminal cocci Bifidobacteria and Escherichia coli decreased, while the Pap bacteria increased. A recent review of 26 studies showed that depression patients may be characterized by high abundance of pro-inflammatory bacteria (such as Enterobacteriaceae and Desulfovibrio) and low SCFAs producing bacteria (such as Faecalibacterium) (Simpson et al., 2021). Stevens et al. (2018) compared the intestinal microflora of patients with depression and healthy people, and found that Faecalibacterium and Clostridium XIVa of patients with depression were significantly reduced at the level of genus. Moreover, there are few studies on the correlation between SCFAs and clinical depressive symptoms. Therefore, the impact of drugs on the gut microbiota was excluded in the current study, and patients with first-episode depression were selected for comparisons with the control group (C group) in view of investigating the changes in gut microbiota and metabolite SCFAs and analyzing the correlation between such changes and the disease, which may provide a new therapeutic approach for the treatment of MDD, such as some kind of microbial-rich probiotics or prebiotics.

## 2. Participants and methods

### 2.1. Participants

A total of 45 patients with first-episode depression who visited the Psychiatry Outpatient Department at the First Hospital of Hebei Medical University from May 1, 2021 to November 1, 2021 were recruited for inclusion in the MDD group. A total of 22 healthy volunteers, whose gender, age, height, and other demographics matched the patients in the MDD group, were recruited into the healthy C group. The inclusion criteria of the MDD group were as follows: (1) patients or relatives who signed the informed consent form; (2) patients determined to have first-episode depression according to the Mini International Neuropsychiatric Interview (MINI), conforming to the ICD-10 diagnostic standard, who had not received systemic treatment with antidepressants; (3) patients aged 16–65; and (4) patients with no severe gastrointestinal diseases. The exclusion criteria of the MDD group were as follows: (1) patients having one or more episodes of mania or hypomania, secondary depression, or recurrent depressive disorders; (2) patients administered other probiotics, nutritional supplements (e.g., vitamin C), ginseng, dehydroepiandrosterone, melatonin, antioxidants, antibiotics, laxatives, or other drugs that may affect intestinal homeostasis within 1 month before inclusion; (3) women who were pregnant or lactating; and (4) those on special diets. The inclusion criteria of the C group were as follows: (1) confirmed as healthy in a physical examination, with mental disorders excluded according to MINI; (2) aged 16–65; (3) having normal blood routines and normal cardiac, hepatic, and renal function; and (4) willing to sign the informed consent form and able to complete the study. The exclusion criteria of the C group were as follows: (1) had been administered drugs likely to affect intestinal homeostasis 1 month before inclusion; (2) having severe gastrointestinal diseases, organic brain diseases, coronary heart disease, hypertension, diabetes, hepatic diseases, metabolic diseases, or obesity (BMI ≥ 30 kg/m^2^); (3) women who were pregnant; and (4) those on special diets (Table 1).

**TABLE 1:** Comparison of demographics between the MDD group and the CG group.

Features	MDD group (*n* = 45)	CG group (*n* = 22)	*t* (*X*^2^) value/*Z*-value	*P*-value
Age (years) M (P_25_, P_75_)	32 (22, 45)	24.5 (24, 27)	1.954	0.051
**Gender (*n*, %)**				
Male	7 (15.6)	4 (18.2)	<0.001	1
Female	38 (84.4)	18 (81.8)		
BMI (Kg/m^2^) M (P_25_, P_75_)	23.89 (19.52, 27.23)	20.77 (18.46, 23.83)	1.896	0.058
Smoking (*n*, %)	8 (17.8)	1 (4.5)	1.232	0.267
Waistline (cm) Mean ± SD	80.08 ± 10.44	78 ± 10.35	0.767	0.446
Disease course (month) M (P_25_, P_75_)	12 (3, 33)	/		
**Family history (*n*, %)**				
Negative	32 (71.1)	22 (100)	6.146	0.013
Positive	13 (28.9)	0 (0)		
**Past history (*n*, %)**				
No	29 (64.4)	18 (81.8)	2.13	0.144
Yes	16 (35.6)	4 (18.2)		
**Dietary habit (*n*, %)**				
Western dietary habit	1 (2.2)	0 (0)	/	1
Eastern dietary habit	44 (97.8)	22 (100)		
Mediterranean dietary habit	0 (0)	0 (0)		

Based on the ICD-10 diagnostic standards, patients conforming to four standards for an MDD episode and presenting two symptoms from symptom groups A and B were diagnosed with a depressive episode. A total of 45 cases were included in the MDD group, but fecal samples were only collected from 35 cases and serum samples from 17 cases due to the pandemic, disqualified sampling, and other reasons. Twenty-two cases were included in the C group. The two groups exhibited no statistical differences in age, gender, BMI, smoking history, waistline, past history, or dietary habits. There were more positive cases of MDD in the family histories of the MDD group than in the family histories of the C group (Table 1). This study was approved by the Ethics Committee of the First Hospital of Hebei Medical University, and all patients signed informed consent forms.

### 2.2. Tools

#### 2.2.1. Hamilton depression scale-24

The Chinese version of the Hamilton depression scale-24 (HAMD-24) scale has high reliability and validity (Sun et al., 2017) and is used as a nurse-administered rating scale for assessing the severity of a patient’s depressive state. Two trained examiners used the scale for a joint examination of the patient, largely in the form of conversation and observation. Following the examination, the two examiners conducted independent scoring and an average was taken to allow for better evaluating the severity of the diseases and the treatment effects. The total scores denote the following: <8 = normal, 8–20 = possible depression, 21–35 = mild or moderate depression, and >35 = severe depression.

#### 2.2.2. Hamilton anxiety scale

The Hamilton anxiety scale (HAMA) also required two trained examiners for a joint examination of the patients, again largely in the form of conversation and observation. Following the examination, the two examiners scored the patients independently and then calculated the average score. The HAMA measures both somatic and psychic anxiety (Zhang, 2005), and the total HAMA scores can effectively reflect the severity of the anxiety symptoms, meaning the scale is used to evaluate the severity of the anxiety symptoms of patients with anxiety and depressive disorders and the effect of any drugs and psychological interventions. According to data provided by the National Psychiatric Scale Collaboration Group, an individual with a total score of >29 is considered to suffer from severe anxiety, while a score of >21 means the patient has significant anxiety, >14 means the patient has anxiety, >7 means the patient possibly has anxiety, and <7 means the patient does not have anxiety.

#### 2.2.3. Somatic self-rating scale

The somatic self-rating scale (SSS) is a self-evaluated psychological symptom scale. The SSS classifies somatic symptoms into three levels: 30–39 for mild levels, 40–59 for moderate levels, and >60 for severe levels. This scale has high reliability and validity, and it can be used in comprehensive hospitals to screen for psychological symptoms in patients (Zhuang et al., 2010), helping the physicians to effectively identify any somatic symptoms and determine the severity of the patients’ illnesses earlier.

#### 2.2.4. Trail making test A

Cognitive impairment is an important cause of work disability in a patient with MDD, and executive functioning is an important part of cognitive functioning (Zhou et al., 2020). The trail making test A (TMT-A) is a frequently used executive function test (Hachinski et al., 2006). It has high reliability and validity, and it is easy to use, highly objective, and only takes a short time to complete.

#### 2.2.5. Ruminative response scale

Rumination is a mode of thinking in which individuals repeatedly concentrate on their own negative emotions and their respective causes (Zhang et al., 2016), constantly and passively focusing on negative information related to themselves. Rumination is a meaningless way of thinking that is difficult to control and stop. It not only causes a negative mood, a sense of frustration and powerlessness, anxiety, and depression but also gives individuals a negative interpretation of the current situation and even affects somatic functioning. The ruminative response scale (RRS) consists of 22 items divided into three dimensions: depressive rumination, brooding, and reflective pondering. The scale is scored from 1 (never) to 4 (always), and the higher the score, the more severe the rumination.

### 2.3. Methods

#### 2.3.1. Collection of demographics and scale evaluation

The patients were recruited based on the inclusion and exclusion criteria. First, a MINI evaluation was conducted to screen for patients with first-episode depression and to evaluate other criteria. Then, the patients and their relatives were informed of matters related to the test, and they then signed the informed consent form. The demographic data of the patients, including age, height, weight, body mass index (BMI), past history, and family history, were collected. An evaluation using the HAMD-24, HAMA, SSS, RRS, TMT-A scales was completed, the questionnaires were collected at the site, and the data were entered into a computer.

#### 2.3.2. Collection of fecal samples

An approximate 5-g fecal sample was collected from the patients within 3 days after inclusion. The fecal samples were placed in sampling boxes and collected to conduct 16S ribosomal ribonucleic acid (rRNA) gene sequencing on the gut microbiota. From each fecal sample, 0.25 g was transferred, and the total DNA was extracted according to the QIAamp PowerFecal DNA Kit instructions (QIAGEN, DE).^1^ The concentration and purity of the extracted DNA were measured using a Multiskan GO full-wavelength microplate reader (Thermo Fisher Scientific, USA), and the DNA integrity was tested via agarose gel electrophoresis. Any DNA that qualified during the test was used for a subsequent test. The primer pairs added with different indexes at both ends were used for polymerase chain reaction (PCR) amplification of the V4 zone in the 16S rRNA gene (515F-806R) of the bacterial samples. The PCR reaction system (20 μl) included 10 μl of KAPA HiFi HotStart ReadyMix (KAPA Biosystems, USA), 2 μl of DNA (around 30 ng/μl), and 1 μl of forward/reverse primers (10 μM). The sequence of the 16S forward primer was 5′-GTGCCAGCMGCCGCGGTAA-3 and that of the reverse primer was 5′-GGACTACNVGGGTWTCTAAT-3. The PCR reaction conditions were as follows: 95°C pre-degeneration was conducted for 3 min before 30 cycles of 95°C degeneration were conducted for 20 s, 60°C annealing was then conducted for 30 s before a 72°C extension was conducted for 30 s, and, finally, a 72°C extension was conducted for 10 min. The PCR products were purified using an AxyPrep PCR Cleanup Kit (Axygen, USA) before being eluted. The concentration was determined using Qubit 3.0 (Thermo Fisher Scientific, USA), and an equal volume was transferred from each sample and mixed to form a sequencing library. The molar concentration and insert fragments of the library were determined and quantified using QSEP100 (BiOptic, China) and ABI7300 fluorescence quantitative PCR systems (Thermo Fisher Scientific, USA), respectively. The library qualified in the test was used for paired-end 150 bp (PE150) sequencing using the Illumina MiniSeq platform.

#### 2.3.3. Collection of blood samples

Untreated blood samples were collected from elbow veins on the morning of the day following inclusion. The sampling test tube was gently turned upside down 4–5 times immediately after collection to mix the samples. After full setting, the samples were allowed to stand for 30 min and then centrifuged for 10 min, with a centrifugal radius of 200 px and a speed of 3,500–4,000 r/min. The supernatant serum was transferred to an enzyme-free Eppendorf tube and placed in a freezer at −80°C for storage. The test was conducted using an ELISA reagent kit, with the SCFA serum level determined using the double-antibody sandwich method.

#### 2.3.4. Statistical analysis

All data were entered into SPSS 25 software for processing. The quantitative data conforming to the normal distribution were expressed as mean ± standard deviation, with a group *t*-test used for inter-group comparisons. The quantitative data conforming to a skewed distribution were expressed in terms of the median (minimum value and maximum value), with a two-sample rank-sum test used for inter-group comparisons. The qualitative data were expressed as a relative number, and a *X*^2^ test was used for inter-group comparisons. The correlation analysis was conducted using Pearson correlation analysis or Spearman rank correlation analysis, with a *P*-value of < 0.05 regarded as statistically significant.

## 3. Results

### 3.1. Comparison of scale scores between the two groups

Compared with the C group, the MDD group scores were higher in terms of all of the scales (*P* < 0.05) (Table 2).

**TABLE 2:** Comparison of scale scores between the MDD group and the CG group.

Scale/factor	MDD group (*n* = 45) Mean ± SD or M (P_25_, P_75_)	CG group (*n* = 22) Mean ± SD or M (P_25_, P_75_)	*t*-value/*Z*-value	*P*-value
HAMD-24	29 (26, 35.50)	2 (2, 3)	6.636	<0.05
HAMA	22 (18, 28)	1 (0, 3)	6.624	<0.05
SSS	49.69 ± 10.12	21 (20, 22.50)	6.619	<0.05
TMT-A	45 (32.03, 60.38)	30.40 ± 7.60	3.739	<0.05
Total RRS scores	60.13 ± 14.30	24 (22, 28)	6.603	<0.05
RRS-depressive rumination	29.33 ± 7.73	12 (11, 15)	6.485	<0.05
RRS-brooding	14 ± 3.64	5 (5, 6)	6.595	<0.05
RRS-reflective pondering	12.76 ± 3.76	5 (5, 6)	6.429	<0.05

### 3.2. Comparison of the gut microbiota test results

#### 3.2.1. Analysis of similarities

**FIGURE 1:**
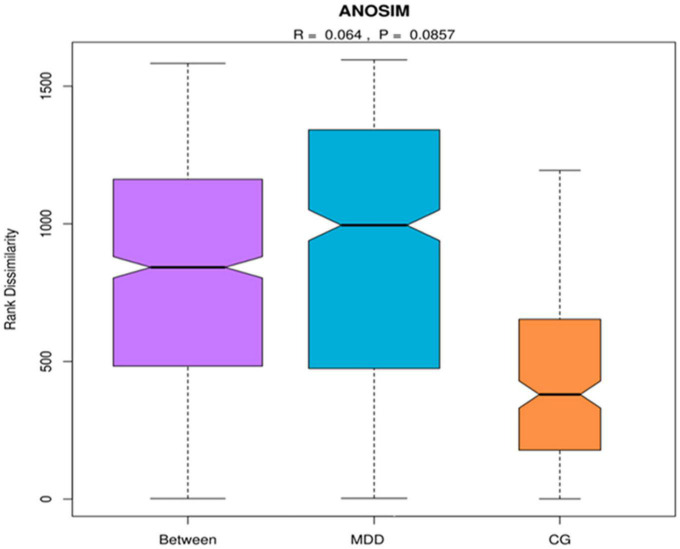
Analysis of similarities. MDD group refers to the major depressive disorder group, and C group refers to the healthy control group. When R ∈ (–1, 1) and *R* > 0, there was a significant inter-group difference, and when *R* < 0, the intra-group difference was higher than the inter-group difference.

An analysis of similarities is a non-parameter test used to test whether the inter-group (two or more groups) differences are significantly higher than the intra-group differences to assess whether the grouping is significant. First, the distance between two samples was calculated using the Bray–Curtis algorithm, and the distances were arranged in ascending order. The results indicated that *R* = 0.064 > 0 (Figure 1).

#### 3.2.2. Analysis of sequencing depth

**FIGURE 2:**
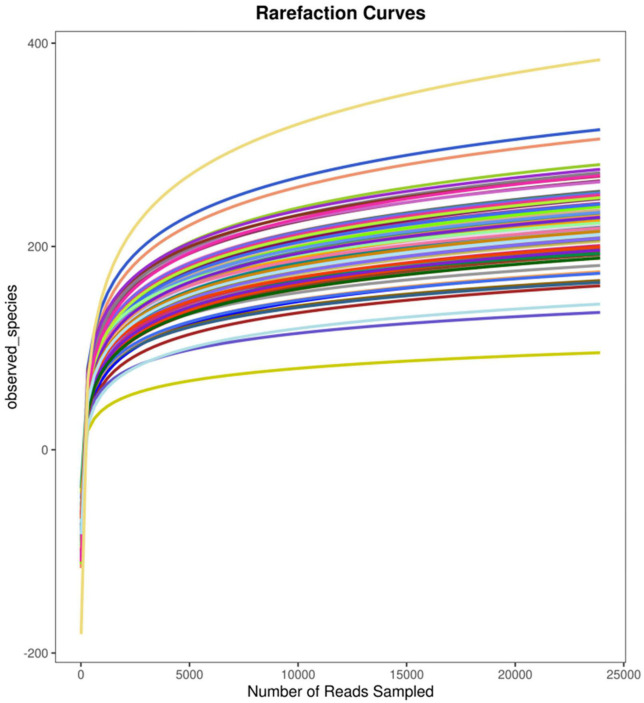
Simpson rarefaction curve.

Due to the pandemic, valid fecal samples were collected from only 57 participants (35 samples from the MDD group and 22 from the C group). To examine the sequencing depth, random sampling was performed for the sequences of each sample, and a curve (the rarefaction curve, which tends to be flat) was plotted using the number of sequences sampled and the figures they represented (Figure 2).

#### 3.2.3. Structural analysis of intestinal microorganisms

##### 3.2.3.1. Analysis of the alpha diversity index of gut microbiota

**FIGURE 3:**
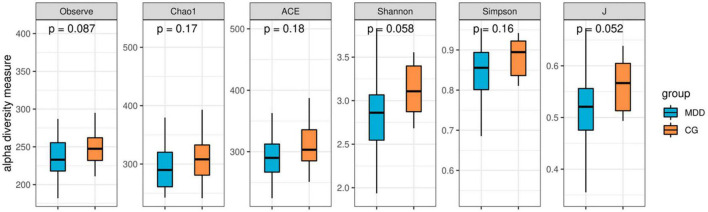
Box plot of α diversity index.

As indicated in the analytical comparison of the gut microbiota alpha (α) diversity index in the MDD group and the C group, the abundance (abundance-based coverage estimator, Chao1, observed species), diversity (Shannon and Simpson), and uniformity (*J*) reflecting α diversity exhibited no statistically significant differences (*P* > 0.05; Figure 3).

##### 3.2.3.2. Analysis of beta diversity of gut microbiota

**FIGURE 4:**
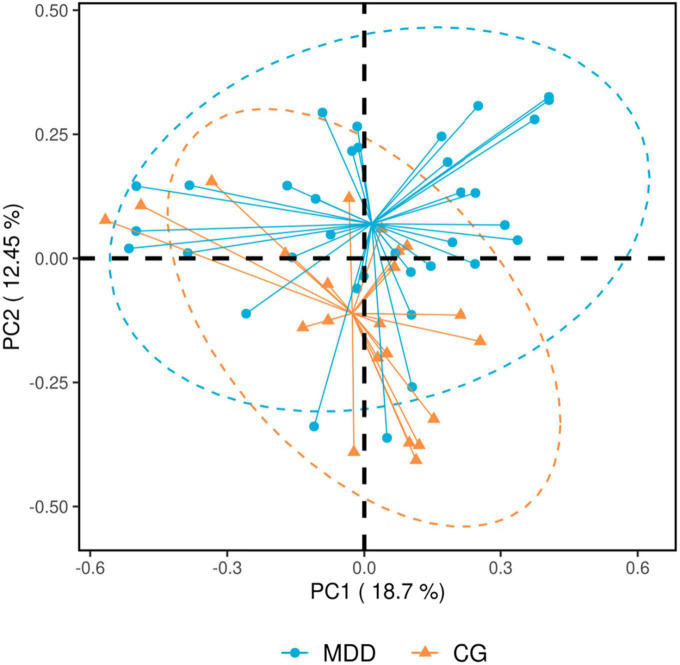
Principal component analysis results. Each spot in the figure represents a sample. The blue spots refer to the major depressive disorder group (MDD group), and the orange spots refer to the healthy control group (C group). The closer the distance between spots, the more similar the samples. Principal component analysis is sequencing analysis based on the species abundance matrix of samples, with the abscissa axis and the ordinate axis representing the contribution rate of principal component 1 (PC1) and principal component 2 (PC2), respectively.

The principal component analysis (PCA) for beta (β) diversity in the MDD group and the C group suggested that the β diversity of the MDD group was higher than that of the C group (*P* = 0.001 < 0.05; Figure 4).

#### 3.2.4. Analysis of dominant bacteria

##### 3.2.4.1. Dominant bacteria at the phylum level

**FIGURE 5:**
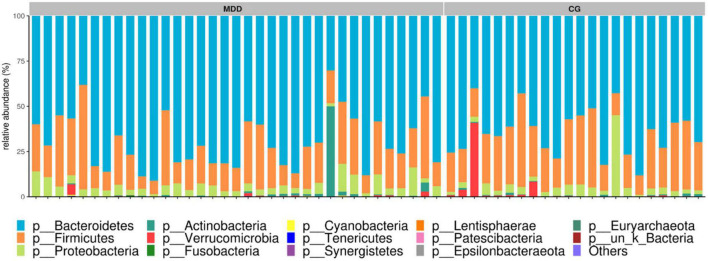
Component analysis of sample species at the phylum level.

As identified in the sequence analysis of the MDD group and the C group, at the phylum level, the gut microbiotas of the two groups belonged to 14 bacterial phyla, and mainly four phyla: *Bacteroidetes*, *Firmicutes*, *Proteobacteria*, and *Actinobacteria*. A comparison between the two groups in terms of the abundance of the four major bacterial phyla revealed no significant differences (*P* > 0.05; Figure 5).

##### 3.2.4.2. Dominant bacteria at the genus level

**FIGURE 6:**
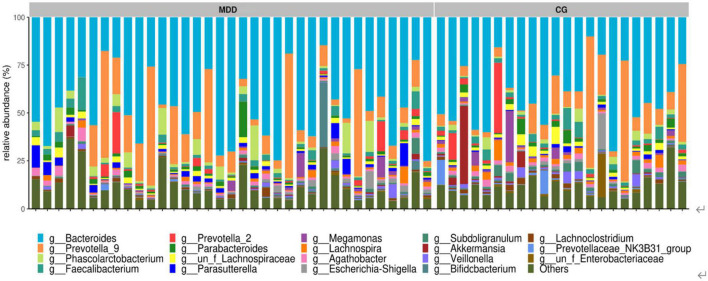
Component analysis of sample species at the genus level.

A total of 293 genera were identified in the samples of the two groups, mainly four bacterial genera: *Bacteroides*, *Prevotella*-9, *Phascolarctobacterium*, and *Faecalibacterium*. A comparison of the four bacterial genera revealed no significant difference between the two groups (*P* > 0.05; Figure 6).

#### 3.2.5. Analysis of differences between key bacterial genera

At the genus level, the results of the analysis of inter-group differences in zone V3–V4 of the gut microbiota 16S indicated that the abundance of *Akkermansia*, *Megamonas*, *Prevotellaceae_NK3B31_group*, *Lachnospira*, *Subdoligranulum*, *Blautia*, and *Streptococcus* in the MDD group was significantly lower than that in the C group, and that the abundance of *Parasutterella* in the MDD group was significantly higher than that in the C group (*P* < 0.05; Table 3).

**TABLE 3:** Comparison of abundance of key bacterial genera at genus level.

Bacterium	MDD group (*n* = 35) M (P_25_, P_75_)	CG group (*n* = 22) M (P_25_, P_75_)	*Z*-value	*P*-value
Akkermansia	0.06 (0, 0.21)	0.38 (0.42, 0.82)	2.374	0.018
Megamonas	0.39 (0.03, 1.11)	0.80 (0.28, 1.74)	2.156	0.031
Lachnospira	0.75 (0.42, 1.41)	1.88 (1.18, 2.69)	3.221	0.001
Parasutterella	1.60 (0.60, 2.89)	0.58 (0.32, 1.33)	2.754	0.006
Prevotellaceae_NK3B31_group	0.14 (0.04, 0.28)	0.50 (0.24, 0.76)	3.830	<0.05
Subdoligranulum	0.46 (0.16, 0.93)	1.32 (0.93, 3.32)	4.238	<0.05
Blautia	0.28 (0.13, 0.43)	0.47 (0.35, 0.65)	2.828	0.005
Streptococcus	0.05 (0.03, 0.10)	0.13 (0.09, 0.35)	3.609	<0.05
Dialister	0.21 (0.11, 0.47)	0.69 (0.31, 1.24)	3.410	0.001

### 3.3. Analysis of the correlation between key bacterial genera and clinical symptoms

**FIGURE 7:**
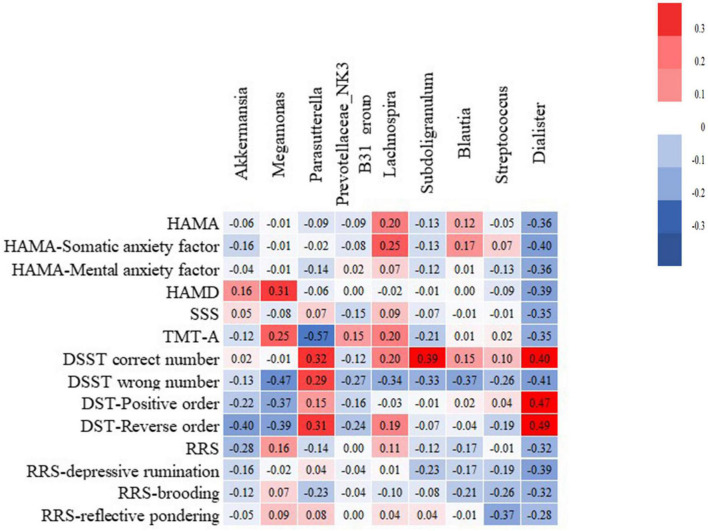
Analysis of the correlation between the abundance of key bacterial genera and clinical symptoms. A positive correlation is red, and the deeper the red, the higher the positive correlation. A negative correlation is blue, and the deeper the blue, the higher the negative correlation. The figure in the box is the correlation index *r*. RRS-Depressive rumination RRS-Brooding RRS- Reflective pondering.

Correlation analysis was conducted in terms of the abundance of gut microbiota at the genus level and the clinical symptom scores (Figure 7). Here, *Dialister* was closely associated with the clinical symptoms and negatively correlated with the HAMA scores, HAMD scores, SSS scores, TMT-A times, total RRS, RRS-depressive rumination, RRS-brooding, and RRS-reflective pondering scores (*r* = −0.36, −0.389, −0.36, −0.389, RRS, RRS-depressive rumination, *P* < 0.05). In addition, *Parasutterella* was negatively correlated with TMT-A time (*r* = −0.570, *P* < 0.05), and *Streptococcus* was negatively correlated with RRS-reflective pondering score (*r* = −0.371, *P* = 0.028).

### 3.4. Comparison of metabolite short-chain fatty acids in the two groups

**FIGURE 8:**
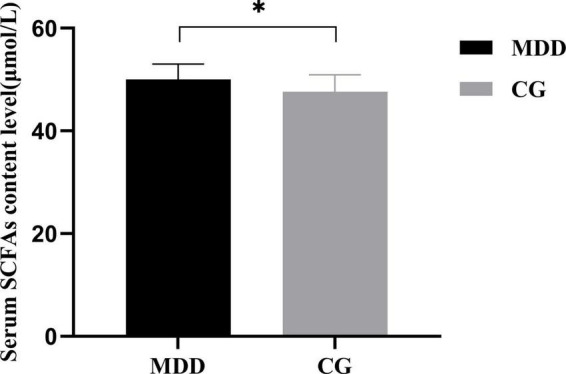
Comparison of serum SCFAs content between the MDD group and the CG group. *P < 0.05.

Due to the pandemic and other reasons, the serum samples were collected from only 39 participants (17 samples from the MDD group and 22 from the C group). A comparison indicated that the serum SCFA content in the MDD group was higher than that in the C group (*t* = 2.322, *P* = 0.026; Figure 8).

### 3.5. Analysis of the correlation between serum short-chain fatty acid content and clinical symptoms

Correlation analysis was conducted in terms of serum SCFA content and clinical symptoms, with the results indicating that the SCFA content was positively correlated with the HAMA scores (*P* < 0.05; Table 4).

**TABLE 4:** Analysis of correlation between serum SCFAs content and clinical symptoms.

	SCFAs	
**Scale/factor**	***r*-value**	***P*-value**
HAMD	0.384	0.128
HAMA	0.584	0.014
SSS	0.249	0.334
TMT-A	0.199	0.445
Total RRS scores	0.258	0.318
RRS-depressive rumination	0.259	0.316
RRS-brooding	0.179	0.493
RRS-reflective pondering	0.197	0.448

### 3.6 Analysis of the correlation between serum short-chain fatty acid content and gut microbiota

**FIGURE 9:**
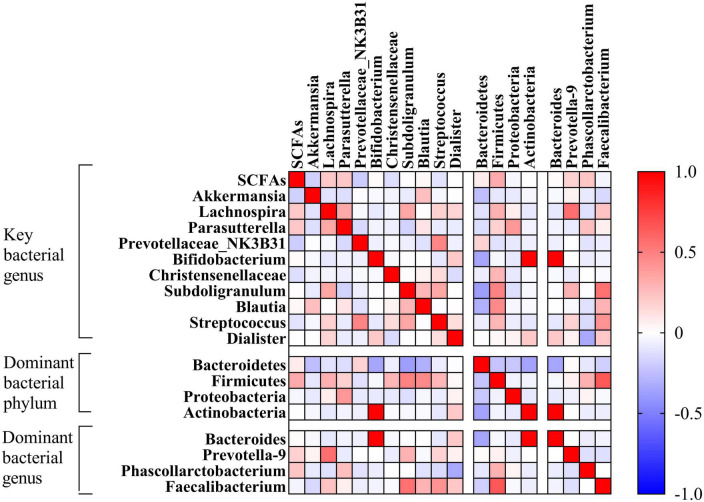
Analysis of correlation between serum SCFAs content and intestinal flora. A positive correlation is red, and the deeper the red, the higher the positive correlation. A negative correlation is blue, and the deeper the blue, the higher the negative correlation. SCFA, short-Chain fatty acids.

Correlation analysis was conducted in terms of serum SCFA content and gut microbiota (key bacteria and dominant bacteria), with the results indicating that the SCFA content was not correlated with any key bacterium or dominant bacterium (*P* > 0.05; Figure 9).

## 4. Discussion

Currently, more than 350 million people suffer from MDD, around 4.4% of the global population (Liśkiewicz et al., 2019), indicating that MDD has a serious social impact. Therefore, a study of the pathogenic mechanism and active exploration of effective therapeutic approaches is urgently needed. As demonstrated in various studies (Chen et al., 2021; Foster et al., 2021), the microbiota-gut-brain axis (MGBA) plays an important role in the pathogenesis of MDD, and changes in gut microbiota are a marked feature in patients with MDD. The metabolites of gut microbiota, SCFAs, have been deemed an important mediator for connecting the MGBA (Dinan and Cryan, 2017; de la Cuesta-Zuluaga et al., 2018).

The similarities analysis and the rarefaction curve of gut microbiota indicated that the grouping and sample size in this study were reasonable. In the structural analysis of gut microbiota, no significant difference was found in the α diversity of gut microbiota between the two groups (*P* > 0.05), but the β diversity index of the MDD group was higher than that of the C group (*P* < 0.05). Lai et al. (2021) also found no difference in the Shannon index, reflecting some diversity between the two groups, while they found that the Fisher index reflecting diversity in the MDD group was lower than that in the C group. This was possibly due to the different number of species included in the studies (Madan et al., 2020; Sanada et al., 2020) or because MDD patients who had been prescribed antidepressants were included, while in our study, the patients included were those with first-episode depression who had not been prescribed antidepressants (Wu Y. X. et al., 2020). Considering the results of other studies, the results of the diversity evaluation of gut microbiota in the MDD group were not consistent (Barandouzi et al., 2020; Sanada et al., 2020) with those of the C group. The analysis of dominant bacteria indicated that at the phylum level, similar dominant bacterial phyla were identified in both groups, and there were no statistical differences in abundance between the two groups (*P* > 0.05). These results were similar to those obtained by Zheng et al. (2016) but were not consistent with those obtained by Jiang et al. (2015) or Chen et al. (2018) as their studies revealed a higher abundance of *Actinobacteria* and *Fusobacteria* in patients with MDD, rather than the *Bacteroidetes*, *Firmicutes*, and other bacteria found in our study. At the genus level, the former four genera of dominant bacteria with similar species existing in both groups were observed (*P* > 0.05). Currently, it remains debatable which dominant bacteria are in the intestinal tracts of patients with MDD and those of healthy people. As the analysis of differences between key bacteria indicated, at the genus level, the abundance of *Akkermansia*, *Megamonas*, *Prevotellaceae_NK3B31_group*, *Lachnospira*, *Subdoligranulum*, *Blautia*, *Streptococcus*, and *Dialister* in the MDD group was lower than that in the C group, and the abundance of *Parasutterella* in the MDD group was higher than that in the C group (*P* < 0.05). In addition, the abundance of *Parasutterella* was negatively correlated with TMT-A time. A significant increase in the abundance of *Parasutterella* was found in patients with MDD following a stroke (Fan et al., 2016), and TMT-A time use reflects executive functioning, meaning this result indicates that an impairment in the executive function of patients with MDD might be associated with an increase in the abundance of some key bacteria. As demonstrated in previous animal tests, an imbalance of gut microbiota, particularly a decrease in *Bacteroides* or an increase in *Proteobacteria*, might be associated with cognitive deficits Shi et al. (2021). Zeng et al. (2019) also found significant changes in the gut microbiota of patients with mild cognitive disorders, mainly an enrichment of *Proteobacteria*, *Synergistetes*, and other bacteria and a decrease in *Epulopiscium fishelsoni*. The result shows that a change in gut microbiota is an important influencing factor for a cognitive change in patients with MDD, implying a possible new approach for the clinical management of cognitive impairment. In addition, the MDD group was found to have a significant decrease in the abundance of butyrate-producing bacteria (*Lachnospira*, *Subdoligranulum*, *Blautia*, and *Dialister*) and acetate-producing bacteria (*Streptococcus*). Butyrate affects the effect of anti-inflammatory and intestinal mucosa repair (Liang et al., 2021). Acetate can serve as an energy substance for surrounding tissues, regulate fat cell differentiation, and synthesize long-chain fatty acids, cholesterol, glutamine, β-hydroxybutyric acid, and other substances (Peng et al., 2021) that are important components of SCFAs.

According to the correlation analysis, *Dialister* was negatively correlated with the clinical symptoms tested in our study, consistent with the results obtained in a previous study (Barandouzi et al., 2020). The abundance of *Dialister* was significantly reduced in the patients with MDD, and this change still existed even after excluding the confounding factor of antidepressants (Valles-Colomer et al., 2019). Therefore, one of the marked features of the disease is a decrease in butyrate- and acetate-producing bacteria (Nikolova et al., 2021). Furthermore, the amount of *Dialister* was significantly associated with the clinical symptoms of MDD, which indicates that for patients with MDD with an inadequacy of any bacterium, supplementing specific probiotics (e.g., probiotics containing abundant *Dialister*), butyrate-producing bacteria, or acetate-producing bacteria, and other biological preparations may present a new treatment approach.

In fact, SCFAs are the main metabolites that are produced from dietary fibers (Blaak et al., 2020) and other substances that are not easily digested via anaerobic fermentation through intestinal microorganisms in the host body; (Koh et al., 2016) they directly or indirectly participate in the functional regulation of the MGBA (Dalile et al., 2019). Based on the fiber content in a person’s diet, around 500–600 μmol of SCFAs are produced in the intestinal tract every day, and acetate, propionate, and butyrate are the most abundant SCFAs in the human body (Bongiovanni et al., 2021). Some SCFAs are absorbed by colon cells and enter the citric acid circulation of mitochondria to provide energy to the cells. Other SCFAs that do not enter the metabolism of the colon cells are transported to the portal vein circulation as the energy substrate of hepatic cells. Acetate is a substrate for synthesizing cholesterol and fatty acids, and propionate is the precursor substance for the liver to synthesize glucose. Therefore, intestinal microorganisms can send signals to the brain of the host through SCFAs that enter the circulation system (Wu M. et al., 2020). A clinical trial (Skonieczna-Żydecka et al., 2018) found that the amount of fecal SCFAs in patients with MDD was lower than that in the control group. Animal tests have also found that the fecal SCFA content in MDD mice was lower than that of those in the control group (Wu M. et al., 2020), which suggests that a decrease in SCFAs may be associated with the pathogenesis of MDD.

However, in our study, the serum SCFA content in the MDD group was significantly higher than that in the C group (*P* < 0.05), without a significant correlation with the key bacteria or dominant bacteria in the gut microbiota. The results of the previous animal study would appear to conflict with the results of our study in that the abundance of bacteria-producing SCFAs in gut microbiota (mainly butyrate-producing bacteria) significantly decreased tyrate-producing bacteria and acet, but the content of serum SCFA significantly increased. In previous studies, SCFAs were considered a protective factor for organisms since they decrease the production of proinflammatory factors, maintain mucous form, and promote mucous repair (Dalile et al., 2019). The amount of SCFAs in the feces of depressive mice and humans has been found to be decreased in most cases. The inconsistency between our analysis and the results of other studies may be due to the difference in the participants included and the test methods. Studies have demonstrated that SCFAs enable anti-inflammatory action in the intestinal mucosa by activating the G-protein-coupled receptors in the intestinal epithelial cells and immune cells and by inhibiting histone deacetylase (Parada Venegas et al., 2019; Beurel et al., 2020). The participants included in this study were patients with first-episode depression, mostly in the acute episode stage, meaning their bodies were experiencing immune activation, and SCFAs, as an anti-inflammatory protective factor, might increase irritability in the acute stage. It might also be caused by different dietary habits, and no actual measurement of the components of SCFAs. Total SCFA content was determined in the study, but the content of propionic acid and butyrate were not.

Hu et al. (2011) studied the impact of soluble dietary fiber (SDF) on the serum SCFAs of rats fed with high-fat diets, and their results indicated that SDF from different sources had different impacts on the components of serum SCFAs. In most previous studies, the fecal SCFA content was measured, but only the serum SCFA content was measured in our study, meaning it is not possible to demonstrate the consistency between the changes in fecal and serum SCFAs. Furthermore, few serum samples were obtained, which may have led to some bias. Therefore, in future studies, the components of SCFAs in serum and feces should be measured, and the changes in SCFA content during the episode, remission, and other stages in the course of MDD should also be observed dynamically to better understand the causal relationship between SCFAs and the pathogenesis of MDD.

There were a number of limitations in this study. First, the sample size of this study was small (35 fecal samples from the MDD group and 22 from the C group, 17 serum samples from the MDD group and 22 from the C group), and it is still necessary to expand the sample size to further verify the role of gut microbiota and its short-chain fatty acids in the pathogenesis of depression; and the participants included in the study were mostly from Hebei, China, with similar dietary habits. Second, the examination of the gut microbiota was conducted in part using a cross-sectional study, without a dynamic observation of the changes in gut microbiota in the acute stage, remission state, and recovery stage of MDD. Third, the SCFAs in the feces and serum were not tested at the same time, meaning it is not possible to verify whether the change in serum SCFAs also indicates a change in feces. Fourth, the components of the SCFAs were not tested, meaning the results may be less accurate.

In this study, the changes in the abundance and diversity of some gut microbiotas in patients with MDD, specifically the significant decrease in butyrate- and acetate-producing bacteria, were observed. *Dialister*, a key bacterium possibly affecting the pathogenesis of MDD, was found to be closely associated with the clinical symptoms, meaning further studies targeting this bacterium should be conducted. In addition, compared with the C group, the level of SCFA in the serum of the MDD patients was significantly higher. Then, whether *Dialister* is related to the increase of SCFA in serum, and whether it participates in the pathogenesis of depression through its SCFA, will be worth further exploration. In conclusion, this study reveals that in our sample there is a correlation between intestinal flora and depression.

## Data availability statement

The original contributions presented in this study are included in the article/supplementary material, further inquiries can be directed to the corresponding author.

## Ethics statement

The studies involving human participants were reviewed and approved by the Ethics Committee of the First Hospital of Hebei Medical University (20210719). The patients/participants provided their written informed consent to participate in this study.

## Author contributions

CA: conception and design of the research and obtaining financing. SY, YW, and XJ: acquisition of data. LW, YW, and XJ: analysis and interpretation of the data. SY: statistical analysis. SY and LW: writing of the manuscript. CA and LW: critical revision of the manuscript for intellectual content. All authors read and approved the final draft.

## Footnotes

1
https://www.qiagen.com/cn/shop/sample-technologies/dna/genomic-dna/qiaamp-powerfecal-dna-kit
